# Antimicrobial efficacy of chlorine agents against selected oral pathogens

**DOI:** 10.1007/s00784-023-05190-0

**Published:** 2023-08-22

**Authors:** Ulrica Scherdin Almhöjd, Anna Lehrkinder, Ann-Marie Roos-Jansåker, Peter Lingström

**Affiliations:** 1 Department of Cariology, Institute of Odontology, Sahlgrenska Academy, University of Gothenburg, Box 450, SE-405 30 Gothenburg, Sweden; 2 Department of Periodontology, Faculty of Odontology, Malmö University, SE-205 06 Malmö, Sweden

**Keywords:** Chlorhexidine, Chlorine compounds, Dakin’s, Dental caries, Oral disease, Periodontitis

## Abstract

**Objectives:**

Method-dependent comparison of antimicrobial agents’ efficacy against oral pathogens.

**Materials and methods:**

Several sodium hypochlorite solutions (NaOCl)—Perisolv^®^, Carisolv^®^ and Dakin’s solution—were equated with chlorhexidine (CHX) and hydrogen peroxide (H_2_O_2_) against ten oral micro-organisms related to caries and periodontitis using different minimum inhibitory concentration (MIC) and the minimum bactericidal concentration (MBC) techniques. Agents were adjusted to the final 70 mmol/L concentration of active chlorine molecule.

**Results:**

Apart from H_2_O_2_ and the amino acids of Perisolv^®^, all the agents revealed an antimicrobial effect. Agar diffusion test ranked CHX (*p* < 0.05) as the most effective against all ten specimens, followed by the NaOCl of Perisolv^®^ and Dakin’s solution. Correspondingly, in broth microdilution on agar, CHX was the most effective in eradicating micro-organisms at 0.03 mmol/L compared with 2.2 mmol/L of Dakin’s solution. In contrast, the bactericidal concentration of Dakin’s solution was the most effective at 0.2 mmol/L, (*p* < 0.001), followed by Perisolv^®^ (2.14 mmol/L), CHX (2.38 mmol/L) and Carisolv^®^ (3.33 mmol/L) after 5 and 10 min in broth dilution test. In live/dead analysis, 60-min exposure to a 2-fold concentration of agents resulted in two-log *Aggregatibacter actinomycetemcomitans* inhibition by CHX (35 mmol/L) whilst *Streptococcus mutans* was more susceptible, in 0.8 and 8.8 mmol/L, after 10 min to CHX and Dakin’s respectively.

**Conclusion:**

Replacement of CHX with tested hypochlorite agents showed evident potential and promoted rapid antimicrobial effect.

**Clinical relevance:**

Effective antimicrobial agents are crucial in controlling pathogen-induced oral infections increasing clinical possibilities to combat oral biofilms. Additionally, CHX substitution with hypochlorite agents could eliminate CHX’s adverse effects.

**Supplementary Information:**

The online version contains supplementary material available at 10.1007/s00784-023-05190-0.

## Introduction

Oral biofilms, associated with oral diseases including dental caries and periodontitis, comprise a large number of oral micro-organisms [1–3]. A vast community of bacteria is closely related to the occurrence and function of the complex dental plaque that further can lead to the tissue destruction at patient level and outlines oral infections [3–8].

In dental caries, many of the acid-producing bacteria are gram-positive in nature, including *Streptococcus mutans*, *Streptococcus mitis* and *Actinomyces naeslundii* [5, 9], whereas periodontally related species, such as *Staphylococcus aureus*, *Prevotella intermedia*, *Prevotella nigrescens* and *Fusobacterium nucleatum,* are more frequently facultative anaerobes and belong to the phylum of gram-negative bacteria [10, 11]. Collectively, the microbiome is a direct precursor of the induced diseases [2, 6]; however, *Streptococcus mutans* is still the main contributor to the adhesion and dwelling of the dental biofilm as a result of its amyloid formation capacity [5, 12].

Over time, different preventive strategies have been used to reduce the number of micro-organisms and, thereby, the risk of disease [13–15]. Interest has focused on mechanical plaque removal, but a variety of antimicrobial agents have also been introduced [16].

Chlorhexidine is a compound that has been shown to possess bactericidal properties and it works by damaging the outer cell layers of bacterial cells, leading to passive diffusion through the inner layer and the subsequent collapse of the membrane potential and lysis of the cells. Studies have reported that gram-positive cocci bacteria are particularly sensitive to chlorhexidine [17]. However, the rate of this reaction is dependent on the pH of the environment in which it occurs [18]. Against periodontal diseases, chlorhexidine (CHX) is the golden standard amongst oral antiseptics and is therefore frequently used [19, 20]. However, the effect of CHX on dental caries is more uncertain [21]. Additionally, local side-effects are reported, particularly from the use of CHX, including discoloration of the teeth and tongue, as well as taste disturbances [21–23].

Agents such as peroxides, Taurolidine, Triclosan, essential oils and Povidone-iodine have been recommended as substitutes for CHX solutions due to their capacity to penetrate the biofilm mass, resulting in either a bacteriostatic or a bactericidal effect [24–28]. Oxidising agents, such as hydrogen peroxide (H_2_O_2_), are commonly used in clinical settings due to their broad-spectrum antimicrobial activity. H_2_O_2_ is effective against gram-negative bacteria and is considered environmentally and tissue friendly, as it rapidly decomposes into water and oxygen [18]. The main constituent of Dakin’s dental solution is sodium hypochlorite (NaOCl), which has well-known antimicrobial properties due to its action as an oxidising agent [7]. The exact mechanism of action is not fully understood, but it is believed that these agents transfer electrons from the substrate, resulting in substrate’s oxidation and simultaneous reduction of the agent, a process that disrupts and/or cleaves the chemical bonds in proteins, carbohydrates [29] and lipids [17]. At alkaline pH, the dominant form of chlorine is hypochlorite (OCl-), which is known to have a more rapid reaction and greater efficacy against bacterial spores compared with other forms [17]. It is therefore routinely used during endodontic work [30]. In addition to NaOCl’s broad antimicrobial features, it does not stain the tissue and is easy to use [31, 32].

Sodium-hypochlorite-based products with added amino acids, launched at the end of the 20th century, are used in chemo-mechanical caries removal (Carisolv^®^) [33, 34] and in cleansing the periodontal pocket (Perisolv^®^) [35]. These products possess an antimicrobial capacity with a grade [36] comparable to Dakin’s solution [7] or stabilised hypochlorous acid [37]. Apart from other halogens—fluoride (F^-^), bromide (Br^-^) and iodide (I^-^)—in aqueous solutions known to be antibacterial, chloride (Cl^-^) is more compatible with biological enzymatic reactions and therefore has least side effects [38]. On the contrary to the relatively unreactive hydrogen peroxide representing slow reaction kinetics with the biomolecules [18, 39, 40], the oxidative chlorine solutions (NaOCl) are both short-lived and easily turned into tissue friendly salt products [40, 41].

This study aimed to compare the efficacy of currently available chlorine solutions, especially chloramine-containing solutions, with other well-known antimicrobial substances on different oral pathogens. The null hypothesis states that chloramines do not have the potential to act as an antimicrobial agent against bacteria related to caries and periodontitis.

## Materials and methods

**Fig. 1:**
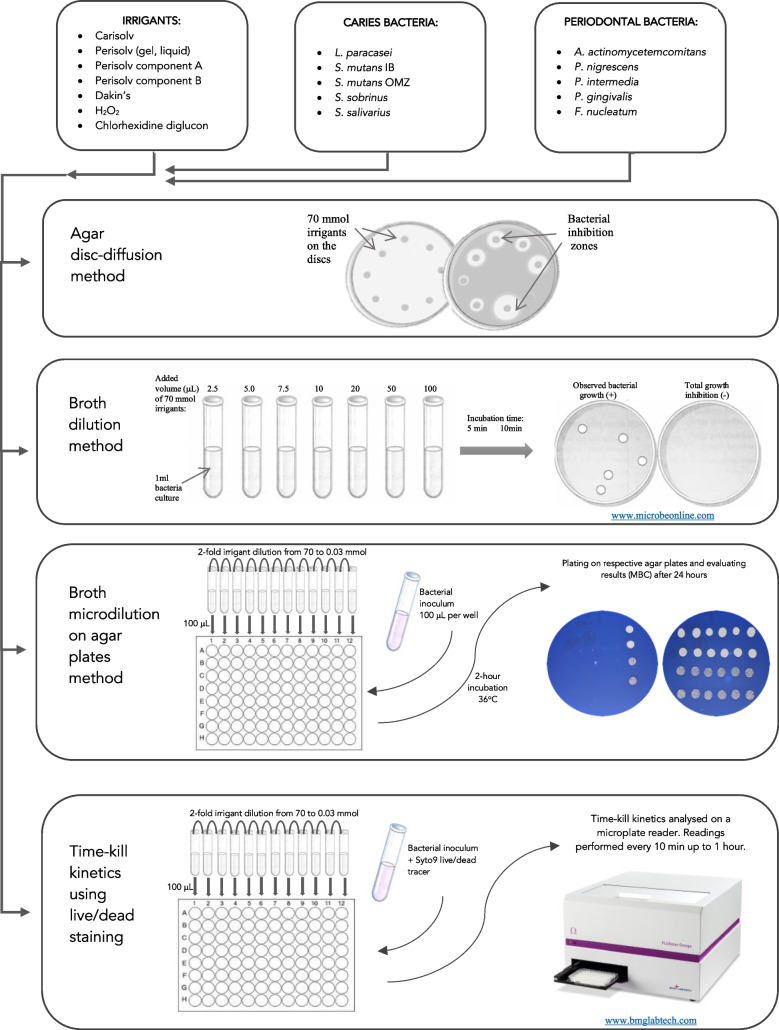
Study design and methods flow chart

Selected irrigants (Table 1) were tested to verify their antimicrobial potency against ten different bacterial strains related to dental caries and periodontitis (Table 2). The efficacy of irrigants was compared using several methods to determine both the minimal inhibitory concentration (MIC) and the minimum bactericidal concentration (MBC) values. A flowchart of methods included in this study is presented in Fig. 1.

**Table 1:** List of irrigants used in the study. The final concentration of chemicals was adjusted regarding the active chlorine at 70 mmol/L (~0.5% of active chlorine) before use

Irrigant	Active part	pH	Abbreviation	Manufacturer
Carisolv^®^ (gel)	-NH_2_Cl	11.0	CAR	RLS Global AB, Gothenburg, Sweden
Perisolv^®^ (gel)	-NH_2_Cl	11.0	PER	RLS Global AB, Gothenburg, Sweden
Perisolv^®^ (liquid)	-NH_2_Cl	11.0	NOG	RLS Global AB, Gothenburg, Sweden
Perisolv, component A (liquid)*	-OCl	11.0	OCL	RLS Global AB, Gothenburg, Sweden
Amino acids of Perisolv, component B (gel)**	-NH_2_	11.0	AAP	RLS Global AB, Gothenburg, Sweden
Dakin’s solution	-(H)OCl	9.0	DAK	APL, Sweden
Hydrogen peroxide solution	-O_2_	4.0	H_2_O_2_	16 911, MERCK KGaA, Darmstadt, Germany
Chlorhexidine diglucon	-HN(C(NH)NH_2_)_2_	7.0	CHX	C9394, MERCK KGaA, Darmstadt, Germany

*Component A, 135 ± 25 mmol l-1; **component B, 53 mmol l-1

**Table 2:** Bacterial specimens used in the study

Bacterial strains	Group	Gram	Abbreviation	Selective agar
*Lactobacillus paracasei* CCUG 32212	Caries	Positive	LBC	Rogosa^A^
*Streptococcus mutans* IB	Caries	Positive	SM-IB	MSB^B^
*Streptococcus mutans* OMZ65	Caries	Positive	SM-65	MSB^B^
*Streptococcus sobrinus* B13	Caries	Positive	SS-B13	MSB^B^
*Streptococcus salivarius* ATCC8618	Caries	Positive	SS-8618	MS^C^
*Porphyromonas gingivalis* OMGS1740	Periodontal	Negative	PG	Brucella^D^
*Prevotella nigrescens* ATCC33563	Periodontal	Negative	PN	Brucella^D^
*Prevotella intermedia* ATCC25611	Periodontal	Negative	PI	Brucella^D^
*Aggregatibacter actinomycetemcomitans* ATCC29522 serotype b, smooth phenotype	Periodontal	Negative	AA	TSB^E^
*Fusobacterium nucleatum* ATCC10953	Periodontal	Negative	FN	Brucella^D^

^A^Difco^TM^ Rogosa SL Agar (Becton and Dickinson, Le Pont de Claix, France)

^B^Difco^TM^ Mitis Salivarius Agar (Becton and Dickinson, Le Pont de Claix, France, supplemented with bacitracin and saccharose)

^C^Difco^TM^ Mitis Salivarius Agar (Becton and Dickinson, Le Pont de Claix, France)

^D^Brucella Agar (Neogen, Lab Acumedic, Heywood, UK)

^E^Tryptic Soy Agar (TSB) ( Sigma-Aldrich, Darmstadt, Germany)

### Preparation of the irrigants

Active chlorine was determined, if applicable, spectrophotometrically (Spectroquant^®^, chlorine test, 1.00599.0001, Merck, Darmstadt, Germany) at 557 nm. The utilised standard curve gave a molar absorptivity, ε: value at 23,000 (mol/L)^-1^*cm^-1^, further used to calculate the total of free and combined chlorine (only active chlorine) in agents using the Beer-Lambert law. The chloramine-based products hold an excess of free chlorine ≈ 80 mmol/L after subtracting the stoichiometric component A (140 ± 7 mmol/L) and component B (53 ± 1 mmol/L amino acids) respectively before mixing. Irrigants containing active chlorine were adjusted with water to the same concentration (70 mmol/L of free or combined chlorine, representing 0.5% w/v of the active substance in DAK, PER and CAR); detailed information is presented in Table 1. Sodium hypochlorite, chlorhexidine and hydrogen peroxide were handled according to EU legalisation from a safety and biocompatibility perspective (the MDR directive in ISO 10993). Furthermore, preparation and handling followed the laboratory’s local restrictions, the material safety data sheet (MSDS) and instructions for use (IFU) when using commercial products.

### Preparation of micro-organisms

The bacterial strains obtained from ATCC (LGC standards, SE) were kept in −80°C at the Department of Cariology, University of Gothenburg, Sweden, before use. Isolated colonies from overnight growth on agar were transferred to phosphate buffer and washed twice. The optical density of each suspension was as assessed at 500 nm and adjusted to 0.5. These standardized suspensions were used for subsequent experiments. Viable bacterial counts (CFU; colony forming units) were obtained after 10-fold dilutions were plated on to respective agar and incubated for 2 days at 37^°^C under anaerobic conditions. Detailed information about bacterial strains is presented in Table 2.

### Agar disc diffusion method

Agar plates were inoculated with the test specimen by swabbing the surface with 3–4 mL of the bacterial suspension. The excess suspension was removed with sterile cotton and plates were dried at 37°C for 30 min to ensure the agar surface was dry. Filter paper discs (Ø, 6 mm) were impregnated with 10 μL of irrigants (70 mmol/L), placed on the agar surface and incubated at 37°C for 24 h in anaerobic conditions (85% N_2_, 10% CO_2_, 5% H_2_). The size of the diameters of the inhibition zones was measured after incubation; all the series were performed in triplicate.

### Broth dilution method

Single volumes (2.5, 5.0, 7.5, 10.0, 20.0, 50.0 and 100.0 μL) of the commercial agents (70 mmol/L) were added to test tubes containing 1 mL of bacterial suspension using a Hamilton syringe (0-50 μL). A droplet of the agent was placed on the neck of the tube until all seven volumes had been added. At an exact time, all the tubes were vortexed for 5 s. After five and 10 min of reaction, bacteria were sampled (25 μL in duplicates) and inoculated on blood agar plates. The plates were incubated anaerobically at 37°C for one to three days. The results were confirmed under the microscope and registered as (-) 100% lethality, total inhibition and (+) where one or more colonies were detected. The reported values represented the minimum volume required to obtain the complete lethality of bacterial cells (see Table 3).

**Table 3:** MBC values for each agent on each bacterial strain

	2.3 Agar disc diffusion (inhibition zone, MV ± SD, mm)								2.4 Broth dilution (μL of agent, 70 mmol/L, that eradicated bacteria after 5/10 min)					2.5 Broth microdilution on agar plates (MBC, mmol/L)					2.6 Efficacy of active chlorine **			
	CAR	PER	NOG	OCL	AAP	DAK	H_2_O_2_	CHX	CAR	PER	DAK	CHX	H_2_O_2_	CAR	PER	DAK	CHX	H_2_O_2_	CAR 5 min	CAR 10 min	PER 5 min	PER 10min
*Lactobacillus paracasei* CCUG 32212	7.0 ± 0.7	6.7 ± 0.2	6.5 ± 0.0	9.3 ± 0.9	6.0 ± 0.0	10.3 ± 0.3	6.0 ± 0.0	14.0 ± 1.0	100/50	50/50	2.5/2.5	20/20	>100/>100	8.8	2.2	2.2	0.14	35	12.49	12.19	12.19	12.19
*Streptococcus mutans* IB	9.8 ± 0.3	7.4 ± 0.8	7.0 ± 0.0	8.5 ± 0.4	6.0 ± 0.0	9.3 ± 0.8	6.0 ± 0.0	15.5 ± 0.4	50/20	50/20	2.5/2.5	50/50	>100/>100	> 70	17.5	4.4	0.14	70	11.96	11.56	11.96	11.56
*Streptococcus mutans* OMZ65	7.7± 0.8	7.0 ± 0.7	6.5 ± 0.0	9.8 ± 1.3	6.0 ± 0.0	11.0 ± 0.0	6.0 ± 0.0	15.8 ± 0.3	100/50	20/7.5	2.5/2.5	50/20	>100/>100	> 70	17.5	4.4	0.07	> 70	12.49	12.19	11.79	11.36
*Streptococcus sobrinus* B13	6.7 ± 0.3	6.7 ± 0.2	6.5 ± 0.0	9.0 ± 0.5	6.0 ± 0.0	9.5 ± 0.0	6.0 ± 0.0	16.3 ± 0.3	100/100	10/10	2.5/2.5	50/50	>100/>100	> 70	n/a	4.4	0.14	70	12.49	12.49	11.49	11.49
*Streptococcus salivarius* ATCC8618	6.8 ± 0.3	6.8 ± 0.2	6.5 ± 0.0	8.8 ± 0.6	6.0 ± 0.0	9.3 ± 0.2	6.0 ± 0.0	12.8 ± 0.6	50/20	20/20	2.5/2.5	50/50	>100/>100	> 70	n/a	4.4	0.03	17.5	13.26	12.86	12.86	12.86
Caries group sum of means*	38.0^d^	34.6^e^	33.0^f^	45.4^c^	30.0^g^	49.4^b^	30.0^g^	74.4^a^	400/240	150/107.5	12.5/12.5	220/190	>500									
*Porphyromonas gingivalis* OMGS1740	10.5 ± 1.5	7.6 ± 1.3	6.5 ± 0.0	9.3 ± 0.9	6.0 ± 0.0	9.2 ± 0.6	6.0 ± 0.0	18.8 ± 0.8	10/7.5	20/7.5	2.5/7.5	50/50	>100/>100	n/a	n/a	n/a	n/a	n/a	11.88	11.75	11.88	11.76
*Prevotella nigrescens* ATCC33563	7.5 ± 1.5	7.5 ± 1.5	9.0 ± 0.0	13.5 ± 0.5	6.0 ± 0.0	9.5 ± 0.0	6.0 ± 0.0	20.7 ± 0.5	20/20	100/20	5/5	50/20	>100/>100	n/a	n/a	n/a	n/a	n/a	11.95	11.95	12.65	11.95
*Prevotella intermedia* ATCC25611	9.3 ± 0.3	7.5 ± 1.0	9.0 ± 2.5	10.3 ± 0.8	6.0 ± 0.0	9.8 ± 0.3	6.0 ± 0.0	19.7 ± 0.5	20/20	20/20	5/2.5	50/50	>100/>100	n/a	n/a	n/a	n/a	n/a	12.20	12.20	12.20	12.20
*Agregatibacter actinomycetemcomitans* ATCC29522	6.8± 0.8	7.1 ± 1.4	6.5 ± 0.0	7.5 ± 0.0	6.0 ± 0.0	7.8 ± 0.6	6.0 ± 0.0	16.4 ± 0.4	5/5	10/2.5	2.5/2.5	20/20	>100/>100	70	> 70	8.8	0.07	> 70	11.45	11.45	12.06	12.06
*Fusobacterium nucleatum* ATCC10953	6.5± 0.0	7.2 ± 0.8	6.5 ± 0.0	13.5 ± 0.5	6.0 ± 0.0	9.0 ± 0.5	6.0 ± 0.0	18.3 ± 1.3	20/50	5/5	2.5/2.5	50/20	>100/>100	4.4	70	4.4	0.03	70	12.46	12.86	11.86	11.86
Periodontal group sum of means *	30.1^e^	36.9^d^	37.5^d^	54.1^b^	30.0^e^	45.3^c^	30.0^e^	94.0^a^	75/102.5	155/55	17.5/20	220/160	>500									
RANKING based on sum of means	4	5	6	2	7	3	7	1	4	2	1	3	5									

*Denote letters, in agar disc diffusion test, indicate significant difference (one-way ANOVA with Tukey-Kramer post hoc test; *p*<0.05) amongst agents for sum of means for caries and periodontal group, same letter indicates no difference

**Active Cl^+^: bacterial cell (log_10_ mol/CFU)

*n/a*, results unavailable due to technical difficulties

### Broth microdilution on agar plates

The ten tested micro-organism cultures, suspended in Mueller Hinton Broth, supplemented with 20.25 mg Ca^2+^ and 11.5 mg Mg^2+^ per litre, adjusted to 0.5 at OD_550_, were distributed (100 μL) into 96-well microtiter plates (ThermoFisher Scientific, UK). The 2-fold dilutions (from 70 to 0.03 mmol/L) of the commercial agents (CAR, PER, DAK, CHX, H_2_O_2_) were prepared and then added to the 96-well microtiter plate in aliquots of 100 μL. After mixing with bacterial suspension, the final concentration ranged from 0.02 to 35 mmol/L. Positive (100 μL Triton, X-100, Sigma-Aldrich) and negative controls (no agent) were included. The plates were then sealed with an adhesive plastic film and incubated for 2 h at 36 ± 1°C. After incubation, 10 μL of each sample was plated on corresponding agar plates (Table 2). The plates were incubated anaerobically for 24 h at 37°C and the results were evaluated. The tests were run in duplicate.

### Calculation of the efficacy of the active chlorine on different bacterial species

For CAR and PER, the reactivity of active chlorine in relation to the number of bacterial cells was calculated. The active chlorine (Cl^+^) was calculated from the molecular concentration (mol/L) in set volumes (L) using Avogadro’s constant, numbers of particles in reciprocal mole (NA=6.022×10^23^/mol). These numbers were compared with the total number of bacterial cells from the established (CFU) resulting in the ratio (Cl^+^/bacterial cell), see Table 3.

### Time-kill kinetics method using live/dead staining

Live/dead staining was used to detect the antimicrobial efficacy of the commercial agents in damaging or killing the bacterial cells in a concentration- and time-dependent manner, a method known as time-kill kinetics. Standardized bacterial suspensions were prepared in the manner mentioned above. The live/dead tracer, Syto 9 and propidium iodide were prepared following the protocol for Invitrogen (Filmtracer live/dead biofilm viability kit L 10316, Invitrogen) and 160 μL of the film tracer mixture was added to 16 mL of each bacterial suspension. Mixed bacterial suspensions were next added to the 96-well microtiter plate in aliquots of 100 μL. The antimicrobial agents were also prepared in the same manner as 2-fold dilutions (70 mmol/L), with the final concentration range from 0.02 to 35 mmol/L in aliquots of 100 μL. Before the test, standard curves for each tested specimen stained with Syto 9 were created. Positive (100 μL Triton, X-100, Sigma-Aldrich) and negative controls (no agent) were included. Kinetics were measured in a microplate reader (CLARIOstar, BMG Labtech, GmbH, Germany, equipped with the CLARIOstar software, 2013) using the Syto 9 dye with an excitation/emission maximum at 480/500 nm for intact cells (green) and 490/635 nm for lysed cells (red) respectively. Readings started directly after adding agents and data were collected every 10 min up to 1 h, at 37°C with rate 2 shaking in between measurements. Tests were performed in triplicate.

### Statistical analyses

The mean values ± SD of each test replicate were calculated. A two-factor ANOVA Tukey’s multiple comparison test was used to compare the difference between the MIC values for the agents for different micro-organisms. *p* < 0.05 was considered statistically significant. Secondly, a two-way ANOVA, Sidak’s multiple comparison test, was used to compare caries and periodontal bacteria when treated with PER and CHX.

## Results

The summarised results are presented in Table 3. CHX was the most efficient antibacterial agent in the disc diffusion test, the microdilution on agar plates and the time-to-kill analysis. In contrast, sodium hypochlorite was more potent in the direct broth dilution method. Chloramines (PER) and (CAR) showed great efficacy in the time-to-kill analysis for *S. mutans* (Fig. 5).

### Agar disc diffusion method

The irrigants displayed distinct antimicrobial characteristics, apart from H_2_O_2_ and AAP (both ranking 7), where no growth inhibition was observed (Table 3). The results were analysed for differences between the agents and between the two groups of bacteria: cariogenic and periodontal. The strongest, statistically significant effect on all bacteria was observed for CHX (ranking 1), followed by OCl and Dakin’s solution. The weakest inhibitory effect was observed for NOG (ranking 6). Overall, CAR exhibited a more substantial inhibitory effect than PER. Moreover, OCL (ranking 2) and PER (ranking 5) were more effective against periodontal, gram-negative bacteria, whilst DAK (ranking 3) and CAR (ranking 4) inhibited cariogenic, gram-positive bacteria more efficiently. In all, CHX was the preferred antimicrobial agent to use between both groups of bacteria, with a significant difference in comparison to all the other agents, *p* < 0.05.

### Broth dilution method

**Fig. 2:**
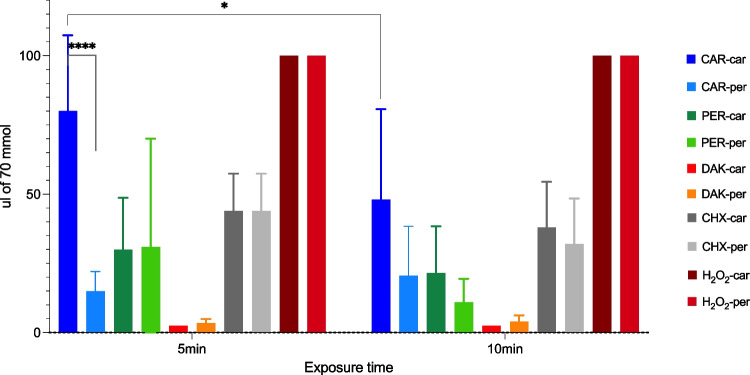
Volume (μL, MV, SD) of 70 mmol agents needed to eradicate five bacterial strains in each group of bacteria (caries and periodontal)

The broth dilution method revealed that the lowest instant killing volume, after 5-min exposure, for DAK was 2.5 μL (all bacterial strains except PN and PG), followed by PER (for FN) and CAR (for AA) 5 μL and for CHX (for LBC and AA) 20 μL (Table 3). Hydrogen peroxide did not inhibit the growth of any tested bacteria. The lowest instant killing effect was more pronounced after 10 min; however, the lethality pattern was similar, regardless of the exposure time. Bacterial groups, caries and periodontal, only differed significantly in response to CAR treatment (two-way ANOVA with Sidak’s multiple comparisons test, *p*<0.0001). Additionally, a significant difference in CAR treatment was observed in cariogenic bacteria between five and 10 min (*p*<0.05), Fig. 2. Significant differences between agents were observed; Table 4 presents details in supplementary material.

### Broth microdilution on agar plates

**Fig. 3:**
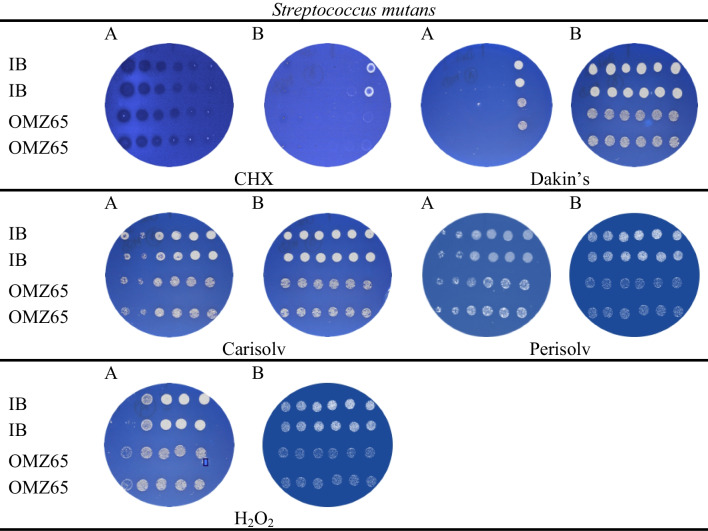
Broth microdilution on agar plates in duplicate. Growth inhibition of *Streptococcus mutans* IB (top two rows) and *Streptococcus mutans* OMZ65 (bottom two rows) on Mitis Salivarius Bacitracin agar by test agents at different concentrations from the left: Plate A: 70, 35, 17.5, 8.8, 4.4, 2.2 mmol L^-1^; Plate B: 1.1, 0.5, 0.3, 0.14, 0.07, 0.03 mmol L^-1^. Drops without colonies (or empty agar surface) equal the MBC value, indicating that the test solution has reduced ≥ 99% of the bacteria. Positive and negative controls worked as expected, not shown

All the agents inoculated in microbial suspensions showed an antimicrobial effect observed on agar plates. Figure 3 presents the representative results obtained for two strains of *Streptococcus mutans*. The MBC values for each agent on each bacterial strain are shown in Table 3. Generally, the lowest MBC value was recorded for CHX (0.03 mmol/L), followed by Dakin’s (2.2 mmol/L) and chloramines (PER, CAR) (2.2–8.8 mmol/L), with H_2_O_2_ being least effective. As expected, due to bacterial structure, the gram-negative bacteria within the periodontal group were more susceptible than the gram-positive, cariogenic bacteria.

### Calculation of the efficacy of the active chlorine on different bacterial species

To calculate the number of single chlorine molecules contributing to the MBC effect of an agent, the stoichiometric ratio of chlorine vs the bacterial cells (1:1) per specimen was calculated. The impact of chloramines (CAR and PER) was similar on SM-IB (11.96), PI (12.20) and PN (11.95), whilst a variation was observed for the remaining bacterial specimens (Table 3). PER emerged as more efficacious, with a lower chlorine/bacteria ratio (≤ 12) than CAR for most tested bacterial species.

### Time-kill kinetics method using live/dead staining

**Fig. 4:**
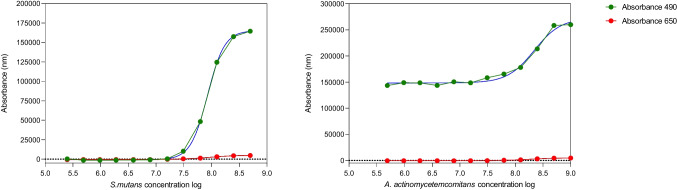
Standard curve of *S. mutans* and *A. actinomycetemcomitans* by Syto 9, live/dead (green 490; red 650nm; blue nonlinear fit curve)

A standard curve of bacterial growth was produced for each tested bacterial species for the live/dead analysis with Syto 9 (490:650 nm), with the absorbance of the living cells at 490 nm that increased over time and the absorbance at 650 nm for lysed cells (Fig. 4).

**Fig. 5:**
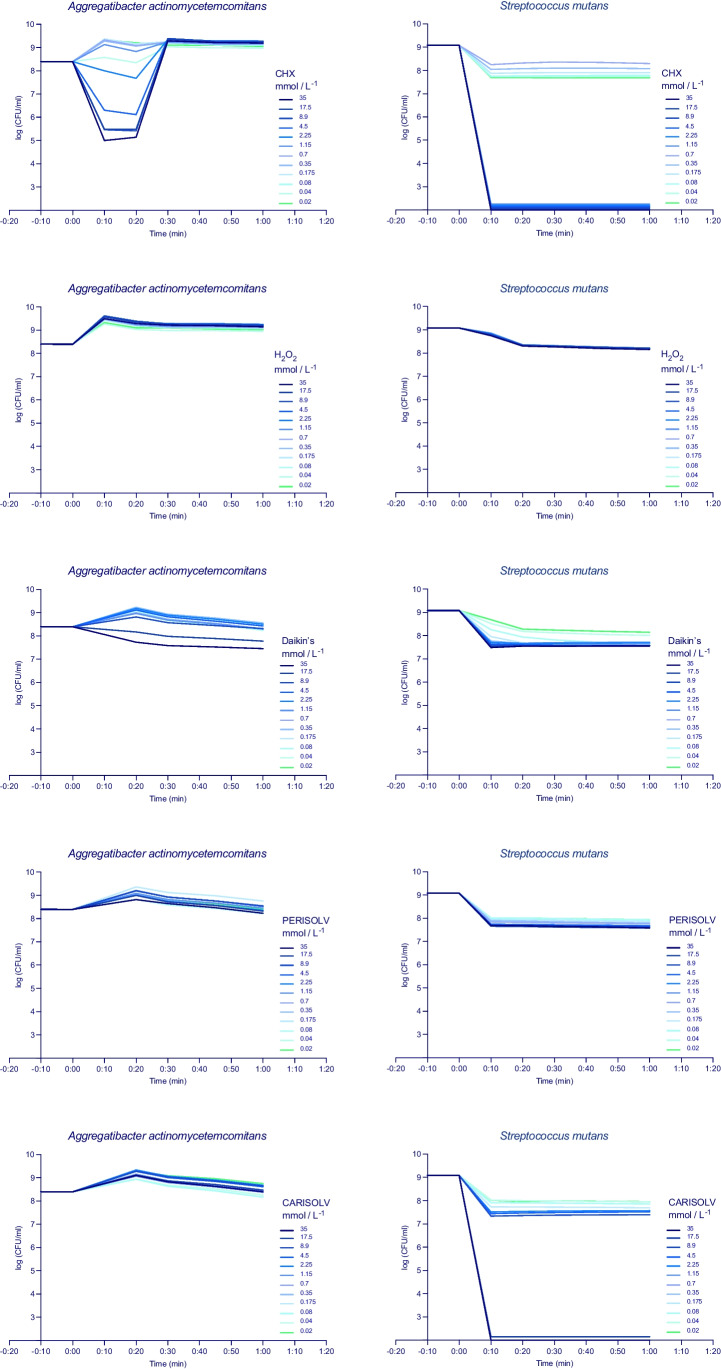
Time-kill curves for all the tested irrigants and one bacterium from each group; AA (periodontal) SM-IB (cariogenic). Twelve 2-fold dilutions are plotted, and the antimicrobial was added at start timepoint and monitored up to 1 h

The time-kill curves for five agents (CHX, H_2_O_2_, DAK, PER, CAR) using *A. actinomycetemcomitans* and *S. mutans IB* are shown in Fig. 5. CHX induced a bacteriostatic effect in all analysed bacteria; the activity depended on the concentration of the agent and differed between bacterial strains. Bactericidal activity, greater than a three log_10_-fold decrease, was obtained with 8.9 mmol/L for AA and 1.15 mmol/L for SM-IB as early as the tenth minute of the test. A similar kinetic pattern was obtained for DAK and PER, with the inhibition of bacterial growth up to 1.5 log_10_. CAR inhibited the growth of *Streptococcus mutans* after 10 min in 17.5 mmol/L. H_2_O_2_ induced minimal growth inhibition in SM-IB and did not affect the growth of AA.

## Discussion

Different antimicrobial agents are regularly applied for dental clinical use, where a reduction in the microbiota leads to a positive outcome in caries and periodontitis treatment. This study compared different commercial chlorine agents with chlorhexidine and hydrogen peroxide using four different MIC methods. The net MIC result favoured chlorhexidine and Dakin’s solution. Furthermore, chloramines were found to have an antimicrobial effect; as a result, the null hypothesis is rejected.

### Agar disc diffusion method

The disc diffusion test established that CHX was the most effective agent, with the diameter of inhibition zones significantly larger than that of other tested agents. These results also agree with other studies showing the more significant impact of CHX on inhibition zones in lower concentrations than NaOCl [42]. On the other hand, CHX and hypochlorite irrigants are reported to produce a similar reduction in bacterial levels during root canal therapy [30], but in vitro testing is highly dependent on its concentration [43, 44]. However, the reaction with CHX is much slower than with the oxidative sodium hypochlorite, which reacts readily in forming short-lived intermediaries [17, 40]. This means that sodium hypochlorite compound is consumed at a higher rate and may explain the better result obtained by CHX in the disc diffusion method that does not deteriorate at the same rate and would therefore be more beneficial under long-term exposure. Moreover, it is vital to consider the molecular weight of an active substance when validating its antibacterial activity. This was found by Müller et al. in 2008 comparing the cytotoxicity and antibacterial activity of commercial agents, where the ranking order was considerably different based on molar concentration (mol/L), apart from mass concentration (w/v) [43]. This was especially evident for reagents with a relatively high molecular weight, such as PHMB and CHX. This was also the basis of this study and the reason why all the concentrations were equaled. Furthermore, CHX was slightly more efficacious on periodontitis bacteria than caries bacteria, which is consistent with the agent being used more frequently in the periodontitis context [24]. However, apart from discolouration and taste disturbances, there are a few cases reporting adverse side effects from CHX. It should be noted that CHX is considered a hidden allergen and might be involved in more cases than recognised [45].

The efficacy of component A in PER, OCL and DAK surpass CHX. These oxidative forms of chlorine act as oxidative agents and gain electrons in the reaction with bacterial proteins, carbohydrates and lipids, thereby disturbing the bacterial cell membrane, most often into lysis [17, 46]. This is a process that resembles the MPO (myeloperoxidase) reaction from the radical oxygen system (ROS) of the immune system, turning HOCl into potent chloramines for the killing of bacterial pathogens in an alkaline milieu [40, 47]. These reactions are considered rapid or short lived [46], as they involve both redox and radical systems [17, 46]. However, rapid agent decomposition may facilitate cell survival during analysis in an agar disc diffusion test, making it less suitable. On the other hand, according to the collision theory in thermodynamics and as stated in the Arrhenius Equation, the rate of a chemical reaction is proportional to the number of collisions between reactant molecules [48]. Moreover, it will increase in a volume (increased surface size) compared with a solid state (disc), as both molecular movement and contact are increased in a solution [48], which is why rapid reaction mechanisms might be more favoured in methods that involve solvents or suspensions.

Regarding the bacterial groups, DAK was more effective in the caries group, whilst OCL (comp A) was more effective in the periodontal group, where there is no marked difference other than the pH value. Comparable results for chloramines, PER and CAR, were observed, with CAR being more effective, especially on caries bacteria, and thus in line with the intent of use. Chloramines have slightly lower oxidative power (oxidation state +½ of chlorine) than both OCL (component A of Perisolv) (+I, Cl^+^) and Dakin’s solution (+I, Cl^+^) and this could explain the lowered inhibitory effect of CAR and PER in comparison with the other aforementioned irrigants. In addition, chloramines are reported to be very unstable and are turned into radicals from the homolysis of N-Cl, thus being consumed rapidly and not effective for a long exposure time [40, 46].

Hydrogen peroxide and AAP (component B of Perisolv) were not effective. AAP (amino acids, pH 10.5) is not a potent antimicrobial agent and, as a result, no growth inhibition was observed. Hydrogen peroxide (oxidation state -I of oxygen, pH 4) is readily decomposed into water if not handled properly and, if so, when it has perhaps evaporated [49], it will have little inhibitory effect. Furthermore, lower concentrations of H_2_O_2_, through bacterial enzymes such as peroxidases, may induce tolerance to lower concentrations of H_2_O_2_ [17]. Even though the concentrations were equalled, the pH of the irrigants was different; OCL (pH 11) and DAK (pH 9), which may influence the results.

It is worth noting that commercially available dental solutions containing CHX and H_2_O_2_ exhibit significantly lower and higher concentrations, respectively, when compared to 70 mmol/L concentrations used in this study. Specifically, CHX solution typically range from 1 to 2 mmol/L (1–2 mg/ml), whilst H_2_O_2_ have concentrations as high as 800 mmol/L (3%). These findings suggest that CHX demonstrates a strong antibacterial efficacy, even at very low concentrations with regard to the commercially available products. On the other hand, and in line with the commercial concentrations, H_2_O_2_ requires relatively high concentrations to achieve a similar effect. This indicates the importance of understanding the mechanism behind the active ingredients, when evaluating the efficacy of dental products which can only be compared if concentrations are equaled.

### Broth dilution

DAK, with its significantly smallest bactericidal volume at both 5 and 10 min of treatment, was followed by PER, CHX and CAR, with H_2_O_2_ the least effective (*p*<0.001). The low reactivity of H_2_O_2_ could be attributed to its decomposition into water [49]. The molecular size and redox reactions of the microbicidal agents may play a role in their effectiveness. Smaller molecules like DAK and PER, which are less sterically hindered than CHX, may disperse more easily in a closed container, leading to a more potent antimicrobial effect. The capacity of chlorine atoms to ‘jump’ between reactants before reaching equilibrium [40] in a hypochlorite solution may also contribute to the longer antimicrobial effect. Taken as a whole, this will favour the oxidative power of hypochlorite solution, for example, compared with CHX. On the other hand, CHX overcame the efficacy of the oxidative H_2_O_2_, which was suspected of being a potent antimicrobial agent after reaction with amines and sulphur components from bacterial cells [17, 47, 50], but this was not observed. Again, the unwanted decomposition of H_2_O_2_ or an effect of the slightly acidic pH of the H_2_O_2_ solution could have hindered these outcomes. Chlorine is perhaps a more potent oxidising agent in bacterial suspensions [51]. Even though CAR was the only agent showing a significant difference in sensitivity between caries and periodontal bacteria and time, significantly smaller volumes of DAK, PER and CHX are needed to obtain lethality.

### Broth microdilution on agar plates

Like previous methods, CHX emerged as most effective, with MBC values below 0.15 mmol/L, followed by Dakin’s solution (2–4 mmol/L) and chloramines (8–17 mmol/L). Hydrogen peroxide was again ineffective. Perisolv had a slightly stronger bactericidal effect than Carisolv, possibly due to added titanium dioxide [51]. Dakin’s solution, which is a buffered solution (NaHCO_3_, pH 9) without amine functions, has a higher oxidative number of the chlorine than the chloramines and this perhaps contributes to the improved effect. In another study, sodium hypochlorite was tested with well-known antibiotics with both a lower antimicrobial effect (MBC; 20mmol/L) and less effect on gram-negative bacteria [52]. In addition, Carisolv and Perisolv are formulated in cellulose gels, which might also be seen as a physical diffusion barrier. Due to their inability to maintain stable anaerobic conditions during preparation and treatment, we did not obtain results for three periodontal bacteria: PG, PI and PN. The remaining AA and FN followed earlier trends of treatment, with CHX and DAK being the most effective.

### Time-kill kinetics method using live/dead staining

Previous studies have investigated the kinetics of CHX on skin infection bacteria using live/dead staining. The results demonstrated that CHX inhibited bacterial count by two logarithmic units at low concentrations (0.125 mmol/L) over the course of several days [53].

As a result, our study monitored bacterial growth exposed to agents over time using Syto 9 dye. We found that the bacteriostatic effect or inhibition using a similar technique with the Syto 9 dye was dependent on their concentration and varied between the tested bacteria. A three-log inhibition change in AA growth was observed during CHX treatment (35-8.9 mmol/L) after 10 min; however, after 30 min, a total recovery was observed. The bactericidal effect of CHX on SM was obtained after 10 min with concentrations of ≥1.15 mmol, whilst lower concentrations resulted in a 1.5-log inhibition change. The periodontal group of bacteria was less sensitive than the caries group and in line with our calculation of single chlorine atoms needed to kill a single bacterial cell at a ratio of 1:1.

Exposure to DAK and PER resulted in a maximum 1.5-log inhibition of *S. mutans*, with no recovery phase. *A. actinomycetemcomitans* responded with a one-log inhibition to the highest DAK concentration and was not affected by PER treatment. Once again, the periodontal group was more resistant to treatments than the caries group. Thus, one conclusion is that chlorine and not chloramines have the same effect on bacteria. A bactericidal effect was observed using Carisolv (chloramine without titanium, ≥17.5 mmol/L) in *S. mutans* after just 10 min. Carisolv appeared to affect the caries bacteria in the same way as CHX. *A. actinomycetemcomitans* growth was not influenced by any CAR concentration.

Hydrogen peroxide treatment resulted in less than a one-log inhibition of *S. mutans* and no inhibitory changes in AA growth pattern. These results may again depend on the decomposition of the molecule into H_2_O [17, 49]. The reactivity of hydrogen peroxide is highly restricted by pH, where alkalinity increases its oxidative effect [46]. In this study, the hydrogen peroxide had an acidic pH.

The initial aim of this study was to evaluate the efficacy of different agents and their performance by diverse antimicrobial methodologies. For this purpose, individual bacterial strains were used. These may not reflect the true situation of a complex oral biofilm as a dysbiotic biofilm would involve many more parameters. It is in future projects a plan to analyse the response of dysbiotic biofilms to examined irrigants using most suitable method.

## Conclusions

Chloramines demonstrated antimicrobial effect in all methods, with convincing results in the kinetic study (CAR) and broth dilution (PER); the null hypothesis is therefore rejected. CHX, on the other hand, is a resilient compound that resists long exposure time, no matter which methodology is used. From the broth dilution methodology in direct contact with a bacterial suspension in the short term, DAK was superior. For this reason, the chosen method must take the mechanism of the substance into account when determining the MIC values for different antimicrobial agents and gram-positive and gram-negative bacteria differed widely depending on both the method and the agent used.

## Supplementary information

ESM 1 (DOCX 18 kb)

## Data Availability

The data that support the findings of this study are available from the corresponding author in response to a reasonability request.
