# Supplementary material for: Antimicrobial efficacy of chlorine agents against selected oral pathogens
Source: Clin Oral Investig. 2023 Aug 22;27(9):5695–707. doi: 10.1007/s00784-023-05190-0 (PMC10492701; doi:10.1007/s00784-023-05190-0)
Supplement: Supplementary file 1 — (DOCX 18 kb) [file 784_2023_5190_MOESM1_ESM.docx]

Table 4. Significant differences (two-way ANOVA with Šídák’s multiple comparison test) between agents and respective bacterial groups (caries, periodontal) were revealed in the broth dilution method after five- and 10-minutes’ treatment.

| Šídák’s multiple comparison test | Mean diff, | 95.00% CI of diff, | Summary | Adjusted P value |
| --- | --- | --- | --- | --- |
|  |  |  |  |  |
| 5min |  |  |  |  |
| CAR-caries vs CAR-periodontal | 65 | 30,14 to 99,86 | **** | <0,0001 |
| CAR-caries vs PER-caries | 50 | 15,14 to 84,86 | *** | 0,0003 |
| CAR-caries vs. PER-periodontal | 49 | 14,14 to 83,86 | *** | 0,0004 |
| CAR-caries vs DAK-caries | 77,5 | 42,64 to 112,4 | **** | <0,0001 |
| CAR-caries vs. DAK-periodontal | 76,5 | 41,64 to 111,4 | **** | <0,0001 |
| CAR-caries vs CHX-caries | 36 | 1,137 to 70,86 | * | 0,0354 |
| CAR-caries vs CHX-periodontal | 36 | 1,137 to 70,86 | * | 0,0354 |
| CAR-periodontal vs H_2_O_2_-caries | -85 | -119,9 to -50,14 | **** | <0,0001 |
| CAR-periodontal vs H_2_O_2_-periodontal | -85 | -119,9 to -50,14 | **** | <0,0001 |
| PER-caries vs H_2_O_2_-caries | -70 | -104,9 to -35,14 | **** | <0,0001 |
| PER-caries vs H_2_O_2_-periodontal | -70 | -104,9 to -35,14 | **** | <0,0001 |
| PER-periodontal vs H_2_O_2_-caries | -69 | -103,9 to -34,14 | **** | <0,0001 |
| PER-periodontal vs H_2_O_2_-periodontal | -69 | -103,9 to -34,14 | **** | <0,0001 |
| DAK-caries vs. CHX-caries | -41,5 | -76,36 to -6,637 | ** | 0,0059 |
| DAK-caries vs. CHX-periodontal | -41,5 | -76,36 to -6,637 | ** | 0,0059 |
| DAK-caries vs. H_2_O_2_-caries | -97,5 | -132,4 to -62,64 | **** | <0,0001 |
| DAK-caries vs. H_2_O_2_-periodontal | -97,5 | -132,4 to -62,64 | **** | <0,0001 |
| DAK-periodontal vs. CHX-caries | -40,5 | -75,36 to -5,637 | ** | 0,0083 |
| DAK-periodontal vs. CHX-periodontal | -40,5 | -75,36 to -5,637 | ** | 0,0083 |
| DAK-periodontal vs. H_2_O_2_-caries | -96,5 | -131,4 to -61,64 | **** | <0,0001 |
| DAK-periodontal vs. H_2_O_2_-periodontal | -96,5 | -131,4 to -61,64 | **** | <0,0001 |
| CHX-caries vs H_2_O_2_-caries | -56 | -90,86 to -21,14 | **** | <0,0001 |
| CHX-caries vs H_2_O_2_-periodontal | -56 | -90,86 to -21,14 | **** | <0,0001 |
| CHX-periodontal vs H_2_O_2_-caries | -56 | -90,86 to -21,14 | **** | <0,0001 |
| CHX-periodontal vs H_2_O_2_-periodontal | -56 | -90,86 to -21,14 | **** | <0,0001 |
|  |  |  |  |  |
| 10min |  |  |  |  |
| CAR-caries vs. PER-periodontal | 37 | 2,137 to 71,86 | * | 0,0259 |
| CAR-caries vs DAK-caries | 45,5 | 10,64 to 80,36 | ** | 0,0015 |
| CAR-caries vs. DAK-periodontal | 44 | 9,137 to 78,86 | ** | 0,0025 |
| CAR-caries vs H_2_O_2_-caries | -52 | -86,86 to -17,14 | *** | 0,0001 |
| CAR-caries vs H_2_O_2_-periodontal | -52 | -86,86 to -17,14 | *** | 0,0001 |
| CAR-periodontal vs H_2_O_2_-caries | -79,5 | -114,4 to -44,64 | **** | <0,0001 |
| CAR-periodontal vs H_2_O_2_-periodontal | -79,5 | -114,4 to -44,64 | **** | <0,0001 |
| PER-caries vs H_2_O_2_-caries | -78,5 | -113,4 to -43,64 | **** | <0,0001 |
| PER-caries vs H_2_O_2_-periodontal | -78,5 | -113,4 to -43,64 | **** | <0,0001 |
| PER-periodontal vs H_2_O_2_-caries | -89 | -123,9 to -54,14 | **** | <0,0001 |
| PER-periodontal vs H_2_O_2_-periodontal | -89 | -123,9 to -54,14 | **** | <0,0001 |
| DAK-caries vs. CHX-caries | -35,5 | -70,36 to -0,6370 | * | 0,0412 |
| DAK-caries vs. H_2_O_2_-caries | -97,5 | -132,4 to -62,64 | **** | <0,0001 |
| DAK-caries vs. H_2_O_2_-periodontal | -97,5 | -132,4 to -62,64 | **** | <0,0001 |
| DAK-periodontal vs. H_2_O_2_-caries | -96 | -130,9 to -61,14 | **** | <0,0001 |
| DAK-periodontal vs. H_2_O_2_-periodontal | -96 | -130,9 to -61,14 | **** | <0,0001 |
| CHX-caries vs H_2_O_2_-caries | -62 | -96,86 to -27,14 | **** | <0,0001 |
| CHX-caries vs H_2_O_2_-periodontal | -62 | -96,86 to -27,14 | **** | <0,0001 |
| CHX-periodontal vs H_2_O_2_-caries | -68 | -102,9 to -33,14 | **** | <0,0001 |
| CHX-periodontal vs H_2_O_2_-periodontal | -68 | -102,9 to -33,14 | **** | <0,0001 |
